# Sustainable Activated Carbon/Poly(vinyl acetate) Composites with Recycled Fillers for Enhanced EMI Shielding Applications

**DOI:** 10.3390/polym18212666

**Published:** 2026-10-28

**Authors:** Mohamed Ouda, Munther Kandah, Aparna Krishna, Jamal Kurdi, Ahmad Abu Haifa

**Affiliations:** 1 Electrical Engineering Department, College of Engineering and Technology, University of Doha for Science and Technology, Doha P.O. Box 24449, Qatar; mohamed.ouda@udst.edu.qa; 2 Chemical Engineering Department, Jordan University of Science and Technology, Irbid 22110, Jordan; mkandah@just.edu.jo; 3 Applied Research, Innovation and Economic Directorate, University of Doha for Science and Technology, Doha P.O. Box 24449, Qatar; 4 Chemical Engineering Department, College of Engineering and Technology, University of Doha for Science and Technology, Doha P.O. Box 24449, Qatar; 5 Queen Rania Al-Abdullah Center for Environmental Sciences and Technology, Jordan University of Science and Technology, Irbid 22110, Jordan

**Keywords:** activated carbon, electromagnetic interference (EMI) shielding, electromagnetic absorption, polymer composites, lightweight shielding materials, waste valorization

## Abstract

Electromagnetic interference (EMI) poses increasing challenges for compact electronic systems and emerging 5G/6G technologies, driving demand for Electromagnetic (EM) absorbers that are lightweight, low-cost, and environmentally sustainable. Conventional absorbers rely on ferrites, magnetic nanomaterials, and multi-step chemical synthesis, all of which consume energy and raw materials, involve hazardous reagents, and generate waste streams of their own. This study shows that two abundant waste streams, recycled tire particles (TP) and crushed glass (CG), can be converted into effective microwave-absorbing composites when combined with activated carbon (AC) in a poly(vinyl acetate) (PVAc) matrix through simple mechanical processing. The microstructure of the composites was examined via scanning electron microscopy (SEM), revealing systematic changes in surface texture, filler dispersion within the polymer matrix, and pore structure with increasing filler content. The complex permittivity, AC conductivity (σ), and loss tangent (tan δ) of the composites were determined from 0.2 to 20 GHz using an open-ended coaxial probe and correlated with their morphology. Dielectric loss was highest where the filler formed a continuous conductive network and decreased once an open pore structure developed, diluting the effective permittivity. Absorption-dominated EMI shielding was evaluated for metal-backed single layers using transmission-line (TL) theory, validated by full-wave simulations in Ansys HFSS. Pure AC composite absorbed weakly at the λ/4 condition but reached −13.5 dB at the 3λ/4 resonance. Incorporating waste-derived fillers improved its microwave absorption performance. At a thickness of 6 mm, the AC–TP composite (ACTP3) achieved a minimum reflection loss of −39.07 dB at 15.75 GHz, while the AC–CG composite (ACCG1) reached −29.4 dB at 16.75 GHz. These results demonstrate that strong absorption-dominated EMI shielding can be achieved using waste-derived carbon/polymer composites fabricated through simple mechanical processing. The approach offers a scalable and sustainable route to EMI shielding materials that avoids magnetic metals, nanomaterials, and chemical synthesis, while diverting industrial and post-consumer waste from landfill.

## 1. Introduction

The increasing density of electronic devices and their higher operating frequencies have increased the risk of electromagnetic interference (EMI) in consumer electronics, automotive systems, aerospace, and defense applications. The development of 5G and 6G technologies, together with the miniaturization of electronic systems, has made electromagnetic compatibility (EMC) requirements more stringent. Therefore, advanced materials are needed to effectively shield and suppress unwanted electromagnetic (EM) radiation and minimize interference between electronic devices [1,2,3]. These EMI shielding materials attenuate incident electromagnetic waves (EMWs) through reflection, absorption, and multiple internal reflections. Absorbing materials contribute to this attenuation through dielectric and magnetic loss mechanisms [4,5]. Unlike reflective shielding materials that merely redirect EM energy, EM absorbers dissipate incident energy as heat through polarization relaxation, conduction loss, and magnetic resonance, making them essential for applications requiring minimal secondary reflection such as radar stealth technologies, anechoic chambers, and densely integrated electronic systems [3,6,7].

Traditional EM-absorbing material systems include ferrites, metal-containing composites, and carbon-based absorbers, each offering distinct advantages and limitations [8,9,10]. Ferrite-based absorbers provide strong magnetic loss through domain wall motion at lower frequencies and natural (ferromagnetic) resonance in the GHz range but suffer from high density and brittleness. Metallic composites can exhibit both dielectric and magnetic losses but face challenges with corrosion, weight, and cost. Carbon-based absorbers, particularly those utilizing activated carbon (AC), have emerged as attractive alternatives due to their low density, high specific surface area, tunable electrical conductivity, and inherent porosity that promotes impedance matching and interfacial polarization [11]. The disordered carbon structure provides multiple loss mechanisms: dipolar polarization from surface functional groups, Maxwell–Wagner interfacial polarization at heterogeneous boundaries, and conduction losses from mobile charge carriers. However, pure carbon-based absorbers typically require large thicknesses to achieve strong absorption due to their moderate permittivity and absence of magnetic response, limiting their applicability in thin, lightweight devices [12].

Recent advancements have increasingly focused on composite strategies that combine carbon matrices with functional fillers to overcome these limitations [13]. Incorporating magnetic fillers introduces magnetic loss and improves impedance matching by increasing permeability, enabling absorption at reduced thicknesses. Dielectric fillers modulate permittivity and enhance interfacial polarization at phase boundaries. Emerging nanostructured systems, such as α-FeOOH nanorods on carbon aerogels, demonstrate enhanced broadband performance through synergistic coupling of multiple loss mechanisms [14]. Several studies have investigated the incorporation of graphene-based nanomaterials, particularly graphene oxide (GO) and reduced graphene oxide (rGO), into polymeric nanocomposites to improve their EMI shielding capabilities [15,16,17]. Other studies have explored smart absorber architectures employing metamaterial concepts [18], multifunctional shielding–thermal-management materials [19], flexible composites with ultralow filler loadings [20], and machine-learning-assisted materials design for accelerated optimization. Despite these advances, most studies rely on commercially produced or synthesized materials, with limited attention to sustainable, waste-derived alternatives. Moreover, the production of nanomaterials and magnetic fillers often involves energy-intensive, multi-step chemical processing, which raises concerns regarding resource consumption and material sustainability. These limitations highlight the need to develop cost-effective absorbing composites that incorporate biodegradable or waste-derived fillers.

Integrating AC with waste-derived fillers offers a promising circular-materials pathway that simultaneously addresses environmental concerns and materials performance requirements. A wide range of agricultural, industrial, and consumer waste streams contain components with potentially valuable electromagnetic properties [2,21]. Agricultural residues, including bagasse, straw, coconut shells, date palm leaves, and pistachio shells, have attracted interest as feedstocks for EMW absorption because of their carbon content and potential dielectric properties [2,22,23,24]. AC produced from agricultural and other waste streams is particularly promising because of its high specific surface area, porous structure, and electrical conductivity [25]. Industrial and post-consumer streams, including end-of-life tire rubber, batteries, and cullet glass from recycling facilities, provide complementary conductive and dielectric fillers [26,27,28]. Utilizing these waste materials not only diverts them from landfills but also reduces the energy and resource demands associated with producing conventional absorber components [29,30].

Although numerous waste-derived microwave absorbers have been reported, most rely on engineered nanomaterials, magnetic constituents or porous architectures to achieve strong absorption, which increases material cost and processing complexity. Filler loading is systematically varied for each filler type to identify optimal formulations and evaluate loading-dependent characteristics. The surface morphology of each formulation is examined via scanning electron microscopy, and the resulting morphological differences are related to the measured dielectric response. The microwave absorption performance of metal-backed single-layer absorbers is then evaluated using transmission-line theory and verified via full-wave simulations in Ansys HFSS. Impedance matching analysis and quarter-wavelength resonance characterization are used to distinguish between fundamental (λ/4) and higher-order (3λ/4) absorption mechanisms.

The specific objectives are to:

- Identify high-performance and sustainable absorber formulations from waste-derived materials;
- Relate the observed surface morphology and filler dispersion to the dielectric behaviour of each composite;
- Evaluate dominant attenuation mechanisms for each filler type;
- Establish composition–thickness design guidelines for X-band (8–12 GHz) and Ku-band (12–18 GHz) applications;
- Provide a comparative study to guide waste-stream selection for next-generation sustainable electromagnetic absorbers.

## 2. Fundamentals of EMI Shielding and EM Absorption

The widespread use of electronic devices, wireless communication systems, and high-frequency technologies has increased the occurrence of EMI, where unwanted EM radiation can interfere with the normal operation of electronic systems [31,32]. Effective EMI mitigation relies on materials that can efficiently attenuate electromagnetic waves (EMWs), thereby minimizing their propagation toward sensitive electronic components. This section outlines the mechanisms that govern EMI shielding, the fundamentals of EM-absorbing materials, and the transmission-line model used in this work to evaluate absorption-based EMI shielding.

### 2.1. EMI Shielding Theory

EMI shielding involves the attenuation of unwanted electromagnetic fields and waves to reduce their coupling with electronic systems and limit electromagnetic radiation in the surrounding environment. The shielding performance of a material is commonly characterized by its shielding effectiveness (SE), expressed in decibels (dB), which represents the reduction in EM energy transmitted through the shielding material. SE is governed by three primary mechanisms: reflection, absorption, and multiple internal reflections. The total shielding effectiveness ($SE_{T}$) is therefore expressed as the sum of the contributions from reflection ($SE_{R}$), absorption ($SE_{A}$), and multiple internal reflections ($SE_{M}$) [33,34]:

(1) $$SE_{T}=SE_{R}+SE_{A}+SE_{M}$$

where $SE_{M}$ can generally be neglected when $SE_{A}$ exceeds 10 dB. Their relative contributions depend on factors such as electrical conductivity, dielectric permittivity, magnetic permeability, material thickness, operating frequency, and structural characteristics [32,35].

**Figure 1:**
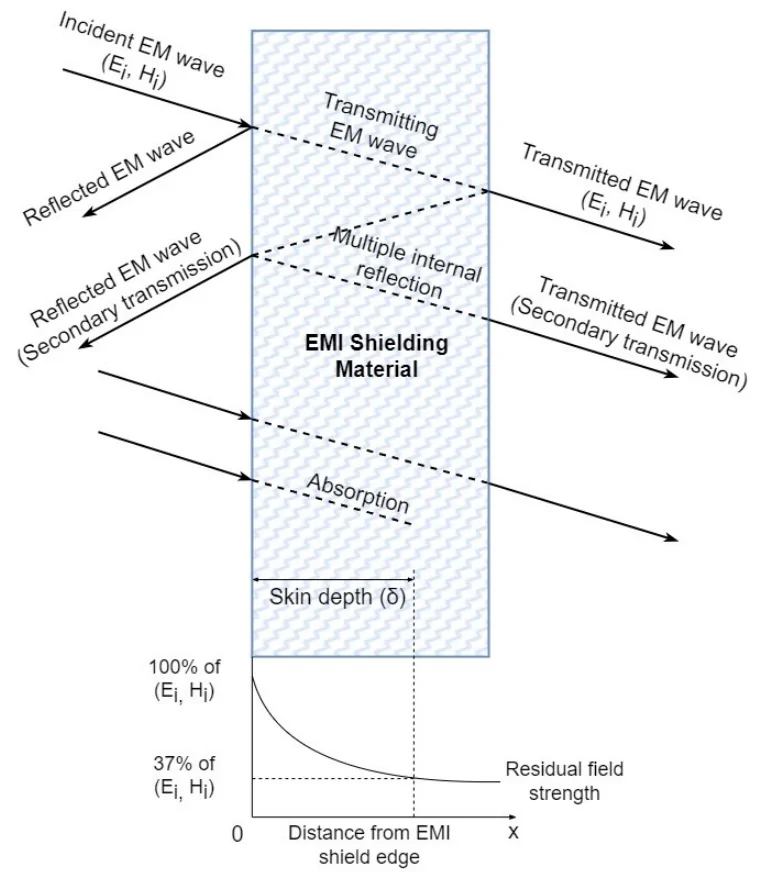
Electromagnetic wave propagation model and the skin depth in EMI shielding materials.

When an incident EMW reaches the surface of a shielding material, a portion of the EM energy is reflected because of the impedance mismatch between the material and the surrounding medium. The remaining energy enters the material and is attenuated as it propagates through the shielding medium. This attenuation can result from electrical conduction, dielectric polarization, and magnetic losses, depending on the composition and electromagnetic properties of the material. Additional attenuation can occur when the propagating wave undergoes repeated reflections at internal interfaces, particularly in heterogeneous, porous, composite, and multilayer structures. These interactions increase the effective propagation path within the material and promote further dissipation of electromagnetic energy. Consequently, only a fraction of the incident EMW reaches the opposite surface and is transmitted through the shielding material, as illustrated in Figure 1 [36].

The contribution of reflection is generally more pronounced in highly conductive materials, where the impedance mismatch at the material–air interface results in strong reflection of incident EMWs. Materials containing dielectric or magnetic lossy constituents can provide substantial absorption by converting electromagnetic energy into other forms, primarily heat, during propagation through the material. To effectively reduce EMI, it is important to develop EMI-shielding materials with high absorption capacity. Such materials dissipate a substantial portion of the incident electromagnetic energy within the shielding medium, thereby reducing the energy reflected back into the surrounding environment. This can help minimize secondary electromagnetic radiation and associated electromagnetic pollution, which may affect nearby electronic devices and biological systems [32]. Therefore, the fundamental principles and mechanisms of absorption-based EMI shielding are discussed in the following subsection.

### 2.2. Fundamentals of EM Absorption Materials

**Figure 2:**
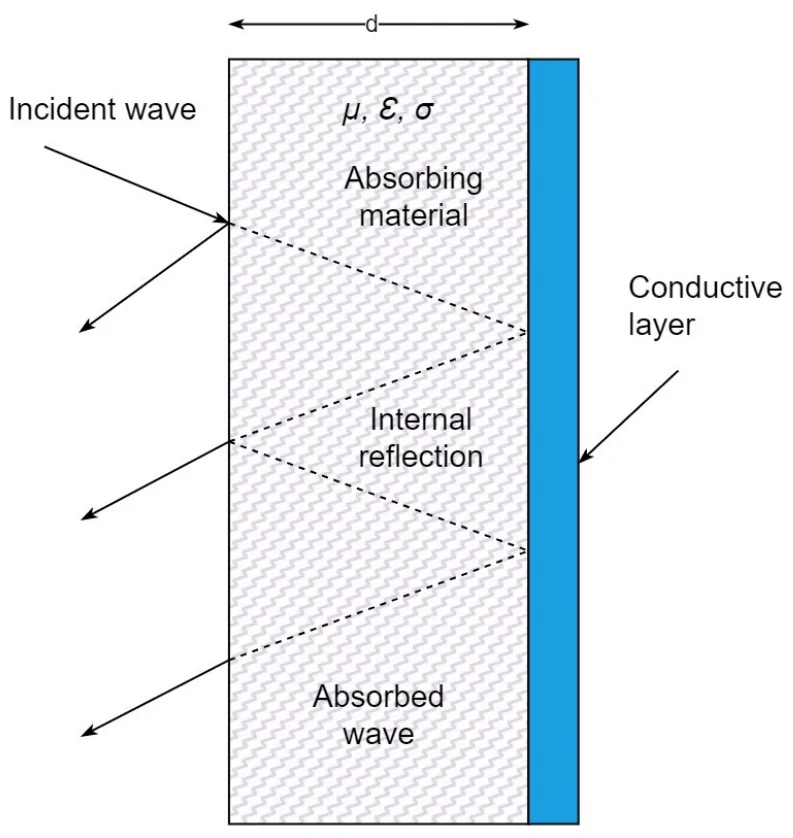
Schematic representation of the mechanism of EM wave absorption and multiple reflections in EM absorber material.

Electromagnetic wave absorbing materials are widely used in EMI shielding, communication systems, electronic devices, and radar technologies to mitigate unwanted electromagnetic radiation. When an incident EM wave encounters an absorber, part of its energy is reflected at the surface, while the remainder enters the material and is either dissipated or transmitted. As illustrated in Figure 2, internal reflections at material interfaces or a conductive backing can increase the propagation path within the absorber, thereby promoting energy dissipation [1]. Effective absorption therefore requires minimal surface reflection and sufficient attenuation of the EM energy entering the material.

Achieving effective absorption depends on two fundamental factors: impedance matching and attenuation capability. Impedance matching facilitates the entry of incident EM energy by minimizing the mismatch between the absorber’s input impedance and the impedance of free space, whereas attenuation capability determines how effectively the penetrated energy is dissipated during propagation. Consequently, high intrinsic losses alone do not guarantee efficient absorption; they must be accompanied by appropriate impedance matching to promote energy penetration and subsequent dissipation. These characteristics are therefore influenced by frequency, material thickness, complex dielectric permittivity (ϵ), complex magnetic permeability (μ), loss tangent (tanδ), and electrical conductivity (σ) [33,34,37].

The complex permittivity and permeability are defined by (2) and (3), respectively [33,34,38]:

(2) $$\epsilon =\epsilon^{\prime }-j\epsilon^{\prime \prime }$$

(3) $$\mu =\mu^{\prime }-j\mu^{\prime \prime }$$

The real parts of permittivity and permeability, $\epsilon^{\prime }$ and $\mu^{\prime }$, describe the ability of a material to store electric and magnetic energy, respectively. The imaginary components, $\epsilon^{\prime \prime }$ and $\mu^{\prime \prime }$, are associated with dielectric and magnetic energy dissipation, respectively [33,34,38]. The dielectric loss tangent, $\tan\delta_{\epsilon }$, represents the ratio of the imaginary to the real component of permittivity and is expressed as in (4) [34].

(4) $$\tan\left|\delta_{\varepsilon }\right|=\left|\frac{\varepsilon^{\prime \prime }}{\varepsilon^{\prime }}\right|$$

Similarly, the magnetic loss tangent, $\tan\delta_{\mu }$, is defined as the ratio of the imaginary to the real component of magnetic permeability [34]:

(5) $$\tan\left|\delta_{\mu }\right|=\left|\frac{\mu^{\prime \prime }}{\mu^{\prime }}\right|$$

When the loss tangent ($\tan\delta_{\epsilon }$ and $\tan\delta_{\mu }$) has a large value, the wave is attenuated as it travels through the material [39]. Conductivity is another important parameter, indicating a material’s ability to conduct electric current or the extent to which it facilitates the movement of charged particles in response to an electric field.

### 2.3. Transmission-Line Model and Reflection Loss

The EM response of a single-layer absorber can be analysed using transmission-line (TL) theory, which provides a framework for determining the input impedance and reflection coefficient of the absorber. Reflection loss (RL) is commonly used to evaluate the reflection characteristics of an absorbing material and represents the amount of incident EM energy reflected from its surface. An RL value of −10 dB indicates that 10% of the incident electromagnetic power is reflected, whereas the remaining 90% is either absorbed or transmitted [38,40,41]. Thus, RL alone cannot distinguish between absorbed and transmitted energy. Although absorption can be determined by considering both reflection and transmission, it can also be evaluated by suppressing transmission. In the method adopted by the United States Naval Research Laboratory (NRL), the absorbing layer is backed by a metal plate, as illustrated in Figure 2, thereby effectively eliminating transmission [42]. Under this configuration, transmission is effectively eliminated, and the non-reflected EM power is dissipated within the absorbing layer. Therefore, an RL of −10 dB corresponds to approximately 90% absorption when transmission is negligible [42].

For a metal-backed single-layer absorber, the input impedance and RL can be expressed as (6) and (7), respectively [34,40,41,43]:

(6) $$Z_{\mathrm{in}}=Z_{0}\sqrt{\frac{\mu_{r}}{\epsilon_{r}}}\tanh\left(i\frac{2\pi fd}{c}\sqrt{\mu_{r}\epsilon_{r}}\right)$$

(7) $$RL=20\log|\Gamma |=20\mathrm{lg}\left|\frac{Z_{\mathrm{in}}-Z_{0}}{Z_{\mathrm{in}}+Z_{0}}\right|$$

where $Z_{\mathrm{in}}$ is the input impedance of the absorber and $Z_{0}=\sqrt{\mu_{0}/\epsilon_{0}}$ is the characteristic impedance of free space. Here, $\mu_{0}$ and $\epsilon_{0}$ denote the permeability and permittivity of free space, respectively, while $\mu_{r}$ and $\epsilon_{r}$ represent the relative permeability and permittivity of the absorbing material. The parameters *f*, *c*, and *d* denote frequency, the speed of light in free space, and absorber thickness, respectively. The reflection coefficient, Γ, corresponds to $S_{11}$ under the conventional one-port measurement configuration.

Thus, as discussed in Section 2.2, the microwave absorption efficiency is governed by the combined effects of impedance matching and electromagnetic attenuation. The degree of impedance matching can be quantified by the normalized input impedance, *Z*, obtained from (8) as [43]:

(8) $$Z=\left|\frac{Z_{\mathrm{in}}}{Z_{0}}\right|$$

When the normalized input impedance approaches unity, the absorber exhibits favorable impedance matching with free space, thereby minimizing surface reflection and facilitating the penetration of EMWs into the material [43]. Conversely, substantial impedance mismatch causes a greater proportion of the incident energy to be reflected at the material surface, limiting the overall absorption performance even when the material possesses considerable dielectric or magnetic loss capabilities. Once EMWs enter the absorber, dielectric polarization, electrical conduction, and magnetic relaxation or resonance processes may contribute to energy dissipation, depending on the material composition and frequency range.

As the EMW propagates through the absorbing material, its amplitude decays exponentially with distance as the energy is converted into heat. This decay is described by the attenuation constant (α), which depends on the complex permittivity, complex permeability, and frequency of the incident wave, and is expressed as in (9) [40,41]:

(9) $$\alpha =\frac{\sqrt{2}\pi f}{c}\sqrt{\left(\mu^{\prime \prime }\varepsilon^{\prime \prime }-\mu^{\prime }\varepsilon^{\prime }\right)+\sqrt{{\left(\mu^{\prime \prime }\varepsilon^{\prime \prime }-\mu^{\prime }\varepsilon^{\prime }\right)}^{2}+{\left(\mu^{\prime }\varepsilon^{\prime \prime }+\mu^{\prime \prime }\varepsilon^{\prime }\right)}^{2}}}$$

Equation (9) shows that the attenuation constant is governed by the dielectric and magnetic properties of the material and the operating frequency. Consequently, achieving an appropriate balance between dielectric and magnetic responses is essential for optimizing impedance matching and internal EM attenuation. For non-magnetic materials ($\mu^{\prime }=1$ and $\mu^{\prime \prime }=0$), (9) reduces to (10), indicating that attenuation is governed entirely by the complex permittivity.

(10) $$\alpha =\frac{\sqrt{2}\pi f}{c}\sqrt{-\varepsilon^{\prime }+\sqrt{{\left(\varepsilon^{\prime }\right)}^{2}+{\left(\varepsilon^{\prime \prime }\right)}^{2}}}$$

Hence, for non-magnetic absorbers, such as the composites studied here, this balance must instead be achieved between energy storage ($\varepsilon^{\prime }$) and dielectric loss ($\varepsilon^{\prime \prime }$), since excessive permittivity increases surface reflection, whereas insufficient dielectric loss limits attenuation. Therefore, effective impedance matching, combined with sufficient EM loss capability, is a fundamental requirement for enhanced microwave absorption. Accordingly, the reflection loss (RL), normalized input impedance (*Z*), and attenuation constant (α) are used in this work to evaluate the absorption-based EMI shielding performance of the composites.

## 3. Materials and Method

### 3.1. Materials

In this study, the recycled tire particles were supplied by Advanced Technology Recycling Materials Co., Zarqa Free Zone, Jordan. The poly(vinyl acetate) binder was supplied by National Paints Factories Co., Ltd., Amman, Jordan. The activated carbon was supplied by Kelin, Henan Province, zhengzhou city, china and the crushed glass was obtained from post-consumer container glass collected locally and reduced manually before sieving.

### 3.2. Methodology

To study the EM-absorbing properties of solid waste materials, composites were prepared by incorporating selected fillers into an AC matrix at specified mass fractions. Activated carbon served as the base material, into which recycled tire particles and crushed glass were incorporated along with diluted poly(vinyl acetate) (PVAc) glue as the binder. Crushed glass and activated carbon were ground into fine particles to improve dispersion and homogeneity within the composite, while tire rubber was shredded and ground to comparably fine particle sizes.

**Figure 3:**
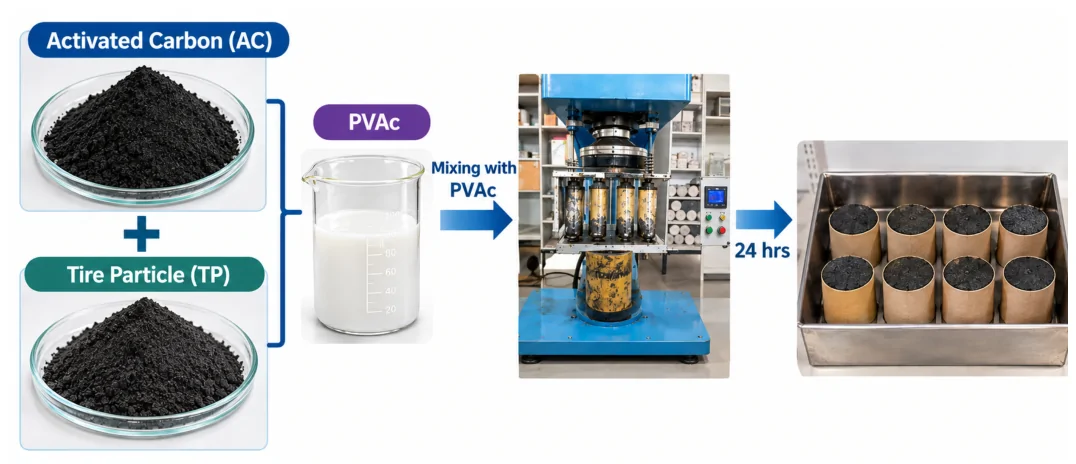
Schematic representation of composite sample preparation.

The fillers were added in specified mass ratios with respect to the activated carbon, as summarized in Table 1. The mixture was thoroughly homogenized and poured into cylindrical molds with dimensions of 60 mm in length and 30 mm in diameter, as shown in Figure 3. The molds were compressed in a hydraulic press at a load of 100 kg over an area of 12.56 cm^2^, corresponding to an applied pressure of approximately 0.78 MPa, and held under load overnight to minimise spring-back and ensure dimensional stability. After demolding, the samples were cured in ambient laboratory air at 25 °C for three days. Substantial moisture evaporation and binder shrinkage occurred during curing, resulting in a significant reduction in sample mass. The cured samples were then weighed and prepared for the analysis of electromagnetic properties.

The fillers were added in specified mass ratios with respect to the activated carbon, as summarized in Table 1. The mixture was thoroughly homogenized and poured into cylindrical molds with dimensions of 60 mm in length and 30 mm in diameter, as shown in Figure 3. The molds were compressed in a hydraulic press at a load of 100 kg over an area of 12.56 cm^2^, corresponding to an applied pressure of approximately 0.78 MPa, and held under load overnight to minimise spring-back and ensure dimensional stability. After demolding, the samples were cured in ambient laboratory air at 25 °C for three days. Substantial moisture evaporation and binder shrinkage occurred during curing, resulting in a significant reduction in sample mass. The cured samples were then weighed and prepared for the analysis of electromagnetic properties.

**Figure 4:**
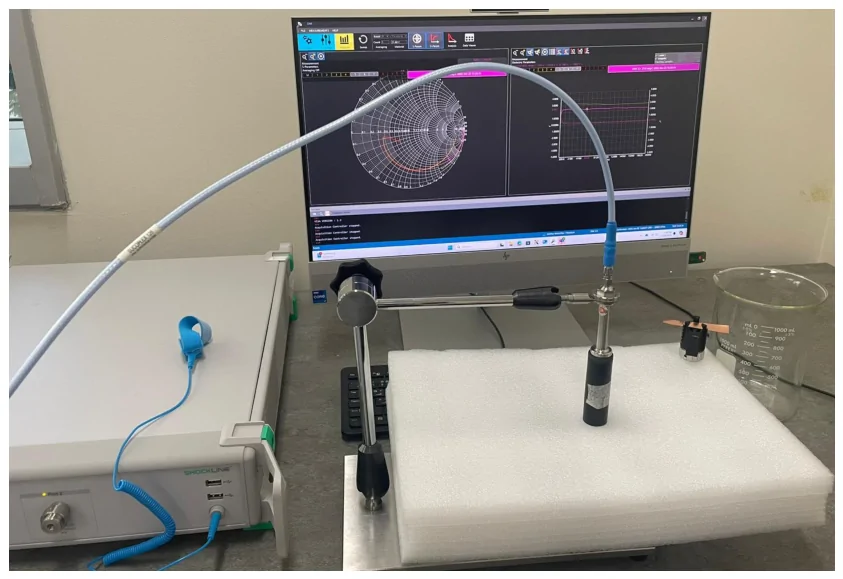
Photograph of the DAK measurement setup showing the open-ended coaxial probe in contact with the proposed composite sample.

Then, the dielectric properties of the fabricated composite samples were analyzed using the open-ended coaxial probe (OECP) method. The open-ended coaxial probe consists of a truncated section of a transmission line. The method involves placing the end of a coaxial transmission line in contact with the material under test (MUT), resulting in an impedance mismatch at the interface. This mismatch causes partial reflection of the incident electromagnetic wave, and the magnitude and phase of the reflection are measured by a vector network analyzer (VNA) over a wide frequency range [44,45]. A dielectric assessment kit (DAK 3.5) was used together with the VNA to extract key parameters such as complex permittivity ($\epsilon^{*}$), tanδ, and σ over a frequency range of 0.2 to 20 GHz [46]. To ensure accurate measurements, the system was calibrated using the Open, Short, and Load (OSL) method with air, a metallic shorting block, and deionized (DI) water to account for variations in the probe and coaxial cable. For each measurement, the probe was placed in firm contact with the smooth, flat surface of the sample, as shown in Figure 4. To account for possible inhomogeneity in filler distribution through the sample thickness, measurements were performed at three different locations on the top surface and three locations on the bottom surface of each sample. The six measurements were averaged to obtain the reported dielectric spectra for each sample.

## 4. Results and Analysis

### 4.1. Material Characterization

**Figure 5:**
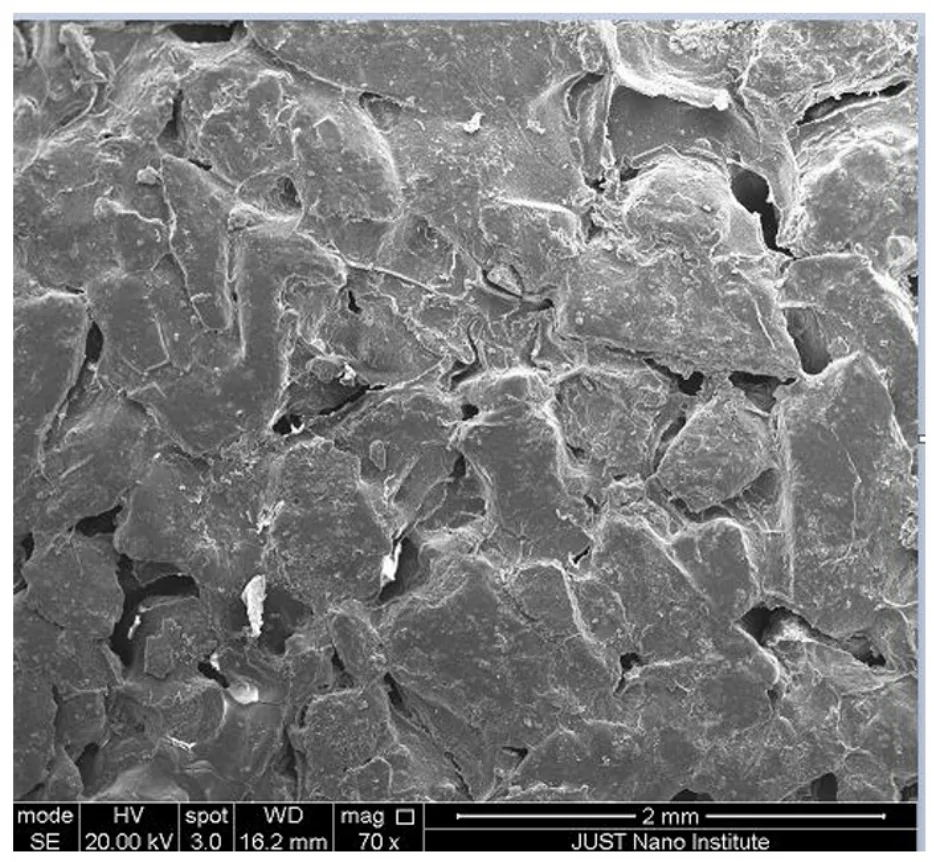
Scanning electron microscopy (SEM) micrograph of sample AC0 (activated carbon + PVAc + water).

The microstructural features of the fabricated composites were examined by SEM to relate their surface morphology to the dielectric response and MWA behaviour. Figure 5 shows the SEM characterization of the reference sample, AC0, containing only activated carbon and PVAc binder. At low magnification, the surface is coarse and densely packed, with large carbon aggregates bound together within the PVAc network. Micro-cracks and small voids are scattered across the surface, left behind by solvent evaporation and binder shrinkage during curing. The densely packed carbon aggregates provide contact points for charge transport, whereas the micro-cracks and voids interrupt the continuity of the conductive network.

**Figure 6:**
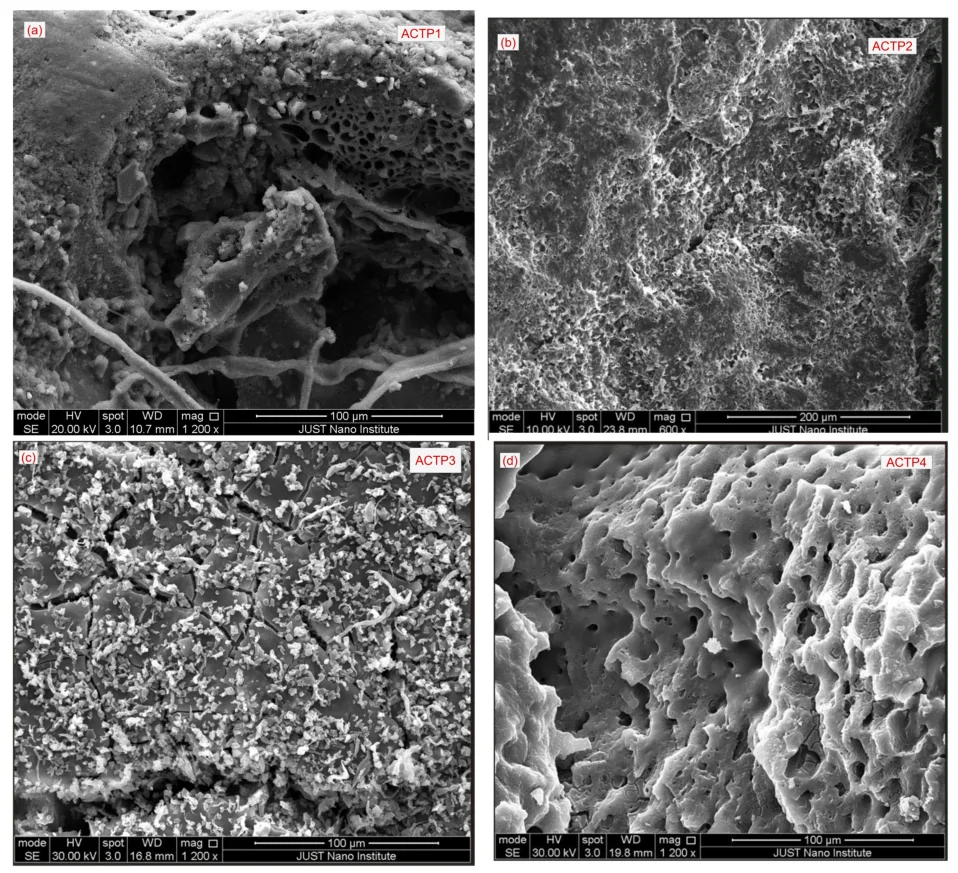
SEM micrographs of samples ACTP1, ACTP2, ACTP3, and ACTP4 (activated carbon + PVAc + water + tire particles).

The SEM images of ACTP1–ACTP4 in Figure 6 show clear variations in surface morphology and particle distribution with increasing recycled tire content. ACTP1 shows a highly heterogeneous fractured surface, with relatively large irregular tire particles embedded in the porous carbon matrix. The carbon-rich regions contain several pores and voids, including areas with a honeycomb-like structure. Sharp interfaces are also visible between the larger particles and the surrounding matrix. In ACTP2, the morphology becomes comparatively finer and more uniformly textured. The surface is rough and densely textured throughout, but noticeably more compact than ACTP1, with no large open voids dominating the observed region. The smaller surface features and more compact structure indicate a change in particle distribution.

ACTP3 displays a distinctly different morphology, characterised by a cracked, plate-like matrix covered with fine particulate features and elongated structures. The particles are distributed heterogeneously across the surface, with localised clustered regions and irregular gaps between adjacent features. The extensive coverage of fine particulate features over the carbon matrix is expected to increase the number of conductive contacts and heterogeneous interfaces, which may enhance conduction and interfacial polarisation losses. ACTP4 exhibits a more porous morphology, with numerous interconnected and closely spaced pores distributed throughout the surface. The pore structure is considerably more pronounced than in ACTP2 and ACTP3, resulting in a relatively open surface with fewer clearly distinguishable larger particulate domains. The high porosity reduces the volume fraction of solid material and is likely to disrupt the continuity of the conductive network, which may lower both the permittivity and the dielectric loss.

The SEM observations demonstrate that increasing recycled tire content modifies the surface texture, filler distribution, and pore structure of the activated carbon-PVAc composite. These morphological changes alter the distribution of interfaces and voids within the composite and may consequently influence its dielectric polarization and microwave attenuation characteristics.

**Figure 7:**
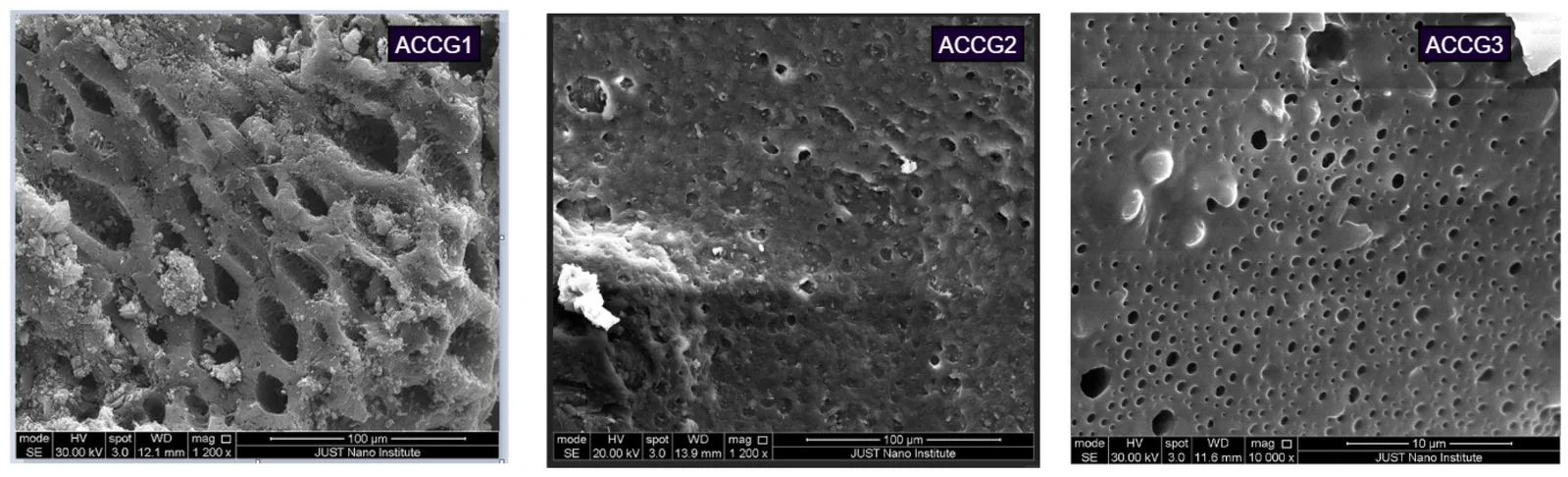
SEM micrographs of samples ACCG1, ACCG2 and ACCG3 (activated carbon + PVAc + water + crushed glass).

Incorporating crushed soda-lime glass introduces insulating inorganic particles into the matrix, altering both its conductivity and pore structure. The SEM images of ACCG1–ACCG3 in Figure 7 show a marked change in surface morphology with increasing glass content. ACCG1 has a clearly open and porous surface with large channels. At this lowest glass loading, the carbon phase remains largely continuous between the pores, which is expected to preserve the conductive network. In ACCG2, this open channel structure is largely absent. The surface is smoother and considerably denser, punctuated only by small isolated pits, with a prominent undispersed agglomerate visible at one edge. Such agglomeration indicates non-uniform filler dispersion, which can locally interrupt the conductive network. ACCG3 shows a more uniform surface with a large number of small sub-micron pores distributed across the surface.

The gradual change from large open pores to a denser surface with smaller pores indicates that the addition of glass changes the pore structure of the material. Since glass is electrically insulating, increasing its content can reduce the continuity of the conducting paths within the carbon matrix. However, the added glass also creates more interfaces between the glass and AC, which can contribute to interfacial polarization. These changes in the surface structure and glass–carbon interfaces can therefore affect the dielectric response of the ACCG composites.

### 4.2. Dielectric Characterization of the Proposed Composite Samples

The dielectric properties of the composite materials with varying percentages of AC and different fillers like tire particles and crushed glass are presented in this section. The performance of these AC-based composites is strongly influenced by the type, distribution, and interactions of the fillers within the matrix. As explained before, AC exhibits remarkable potential for EMW absorption due to its ultra-low density, large specific surface area, and strong dielectric loss capability [47]. To comprehensively understand the EMW absorption behavior of AC, four key dielectric-related parameters such as the real part of permittivity ($\epsilon^{\prime }$), imaginary part of permittivity ($\epsilon^{\prime \prime }$), σ, and tan(δ), are analyzed.

**Figure 8:**
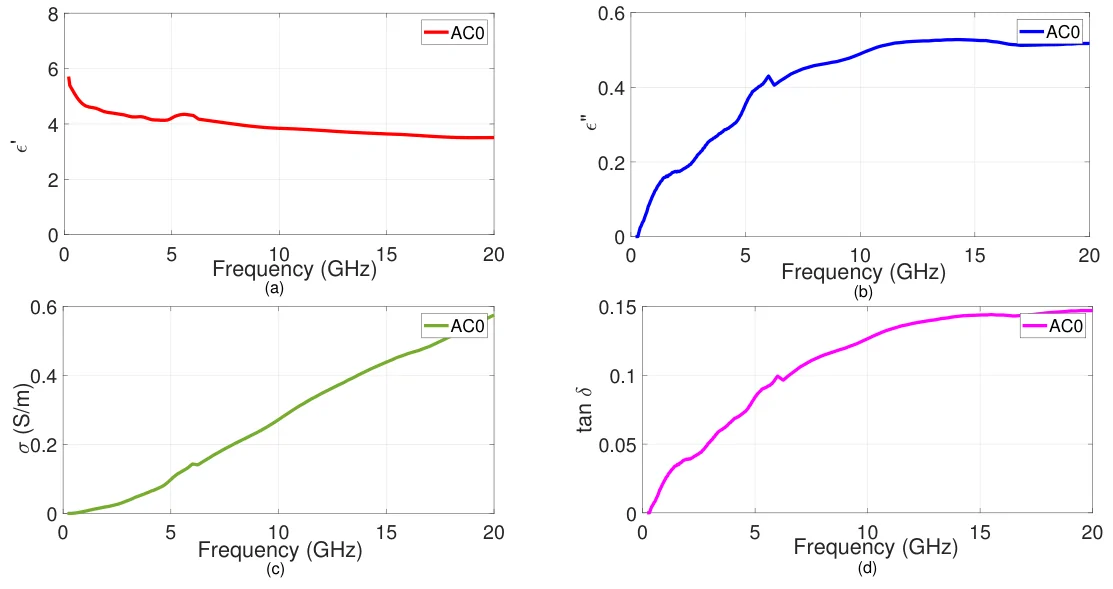
Dielectric Properties of fabricated activated carbon composite (AC0) sample. (**a**) Real Part of permittivity. (**b**) Imaginary Part of permittivity. (**c**) Conductivity. (**d**) Loss tangent.

Figure 8a–d represents the frequency-dependent dielectric properties of the AC0 composite sample. From the Figure, it is clear that the proposed AC-based composite samples exhibit strong frequency-dependent characteristics due to the combined influence of dipolar polarization, interfacial polarization, and conduction behavior. This frequency dependency reflects typical dipolar relaxation, where dipoles and interfacial charges gradually lose the ability to follow the rapidly alternating field at higher frequencies. The relatively high $\epsilon^{\prime \prime }$ indicates strong EM attenuation, which is further supported by the steadily rising conductivity. Conductivity measurements exhibit a clear rising trend with increasing frequency, a behavior characteristic of hopping conduction mechanisms commonly observed in porous carbon-based materials. This behaviour is consistent with the morphology seen in Figure 5, where the carbon aggregates remain in direct contact throughout the PVAc network, providing the continuous pathways required for charge transport. The micro-cracks and voids left by binder shrinkage further contribute internal interfaces at which charge can accumulate. This frequency-dependent conductivity enhances dielectric losses and reinforces the material’s inherently lossy nature by enabling more efficient charge transport pathways at higher frequencies. The effect is further supported by the steadily increasing tanδ, which directly quantifies the material’s ability to dissipate electromagnetic energy as heat, reflecting the combined contributions of interfacial polarization, dipolar relaxation, and conduction loss. Hence, these dielectric characteristics demonstrate that AC0 behaves as a modestly lossy dielectric, providing a useful baseline for broadband microwave absorption and electromagnetic shielding applications.

### 4.3. Activated Carbon and Tire Particle

**Figure 9:**
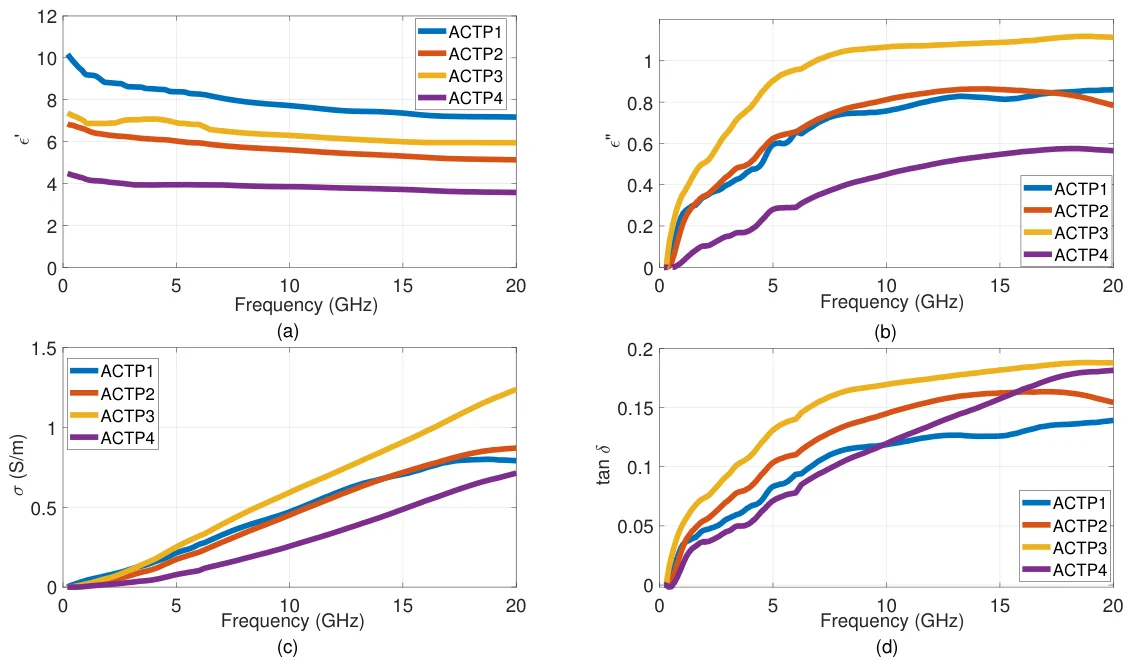
Dielectric Properties of fabricated ACTP composite sample (**a**) Real Part of permittivity. (**b**) Imaginary Part of permittivity. (**c**) Conductivity. (**d**) Loss tangent.

The frequency-dependent dielectric properties of activated carbon-tire particle (ACTP) composites with varying TP ratios (ACTP1–ACTP4) are governed by dipolar relaxation, interfacial polarization, and conduction loss mechanisms as shown in Figure 9. At low frequencies, Maxwell–Wagner interfacial polarization dominates at the heterogeneous interfaces between AC, semi-conductive carbon black within the tire particles, and the insulating rubber matrix, contributing to elevated permittivity values. Then, the $\varepsilon^{\prime }$ decreases with frequency for all samples, reflecting dielectric relaxation where dipoles cannot reorient quickly enough to follow the rapidly oscillating electromagnetic field. ACTP1 exhibits the highest $\varepsilon^{\prime }$, reflecting its activated-carbon-rich composition, in which the extensive carbon surface area and abundant carbon–binder interfaces provide numerous polarization sites [48]. Increasing TP content generally reduces $\varepsilon^{\prime }$, since rubber-based particulates possess lower intrinsic permittivity and fewer polarization sites compared to activated carbon. However, ACTP3 exhibits a slightly higher $\varepsilon^{\prime }$ than ACTP2 across the band. This modest difference is consistent with the dense filler coverage seen in Figure 6, where particulate and elongated features distributed across the matrix introduce additional polarizable interfaces relative to the finer, more uniformly textured surface of ACTP2.

The $\varepsilon^{\prime \prime }$ shows frequency-dependent enhancement for all compositions, indicating increasing energy dissipation from dipolar relaxation losses at lower frequencies and conduction losses at higher frequencies. ACTP3 exhibits the highest $\varepsilon^{\prime \prime }$ and conductivity (σ) of the series, together with the highest loss tangent over most of the measured band. This can be associated with its heterogeneous microstructure and increased interfacial area. This is supported by its SEM morphology, which shows numerous interfaces between the carbonaceous phase, recycled tire-derived constituents, and PVAc binder. Further increasing the TP content (ACTP4) reduces all dielectric parameters. Two factors might have acted together at this composition: the high rubber fraction lowers the intrinsic permittivity of the mixture, while the pronounced open pore network seen in Figure 6 introduces air-filled voids that further dilute the effective permittivity and interrupt the continuity of the conductive carbon network. Both conduction and polarization mechanisms are therefore suppressed, producing a significant drop in overall dielectric performance.

### 4.4. Activated Carbon and Crushed Glass

**Figure 10:**
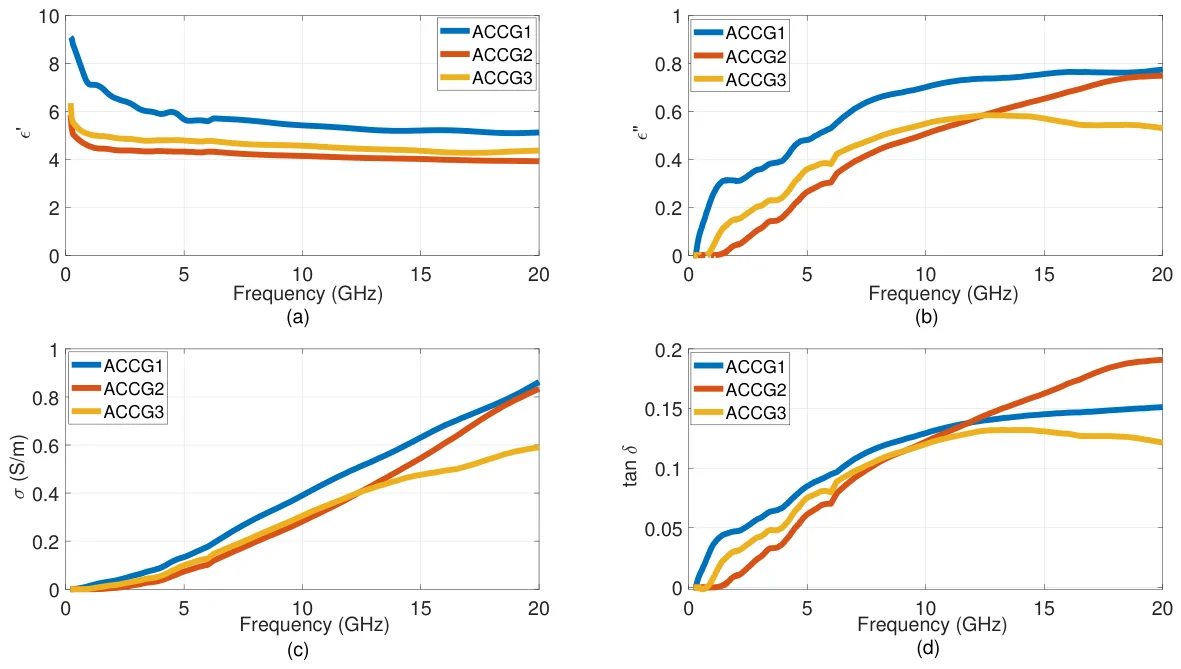
Dielectric Properties of fabricated ACCG composite sample (**a**) Real Part of permittivity. (**b**) Imaginary Part of permittivity. (**c**) Conductivity. (**d**) Loss tangent.

The activated-carbon/crushed-glass (ACCG) composites exhibit frequency-dependent dielectric behavior as shown in Figure 10. The $\varepsilon^{\prime }$ decreases monotonically with frequency across all samples due to classical dielectric relaxation at low frequencies, such as interfacial (Maxwell–Wagner) polarization, space–charge accumulation, and dipolar orientation dominate, but these mechanisms cannot follow the rapidly oscillating field at higher frequencies. Conversely, $\varepsilon^{\prime \prime }$, σ, and tanδ increase with frequency, reflecting enhanced dielectric losses and charge–transport processes.

Across the series, ACCG1 exhibits the highest $\varepsilon^{\prime }$ throughout the measured band, while ACCG2 exhibits the lowest, with ACCG3 lying between the two. This reduction from ACCG1 to ACCG2 is consistent with the morphological change seen in Figure 7, where the open macro-porous channels of ACCG1 give way to a smoother, considerably denser surface in ACCG2. ACCG1 also leads in $\varepsilon^{\prime \prime }$, σ, and tanδ over most of the band. ACCG2 and ACCG3 track closely at low-to-mid frequency, but above approximately 12–15 GHz ACCG2 overtakes ACCG3, leaving ACCG3 with the lowest values of all three by 20 GHz. Since the crushed glass is electrically insulating, its incorporation progressively interrupts the continuous carbon conduction pathway of the unmodified matrix. At the lowest loading, sufficient carbon connectivity is retained for the additional glass–carbon interfaces to contribute meaningful interfacial polarization and scattering. At higher loadings, the disruption of the conductive network outweighs this interfacial contribution, and the overall dielectric loss decreases.

## 5. Analysis of EM Absorption Properties of the Proposed Composites

### 5.1. Pure Activated Carbon Composite (AC0)

**Figure 11:**
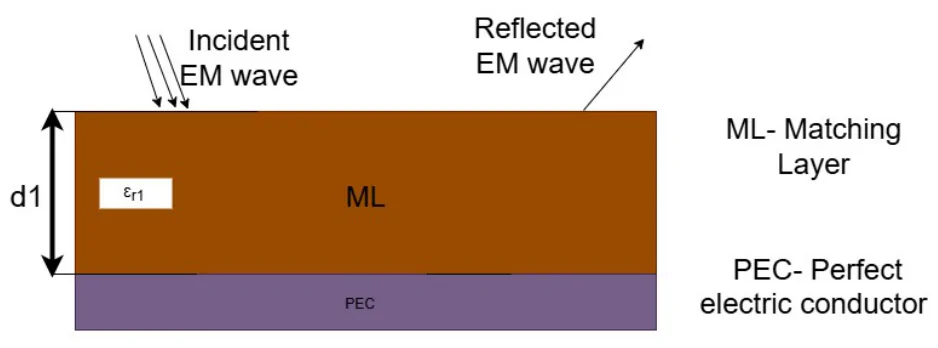
Schematic representation of a single-layer EMW absorbing structure with a backed metal plate.

To analyse the absorption efficiency of the AC composite material, a single-layer absorber backed by a metal plate was modelled as shown in Figure 11, and the RL was computed from the TL equations given in Section 2.3. The complex permittivity measured by the open-ended coaxial probe was used as input over the full 0.2–20 GHz range, and the relative permeability was taken as unity, since the composites contain no magnetic constituents.

**Figure 12:**
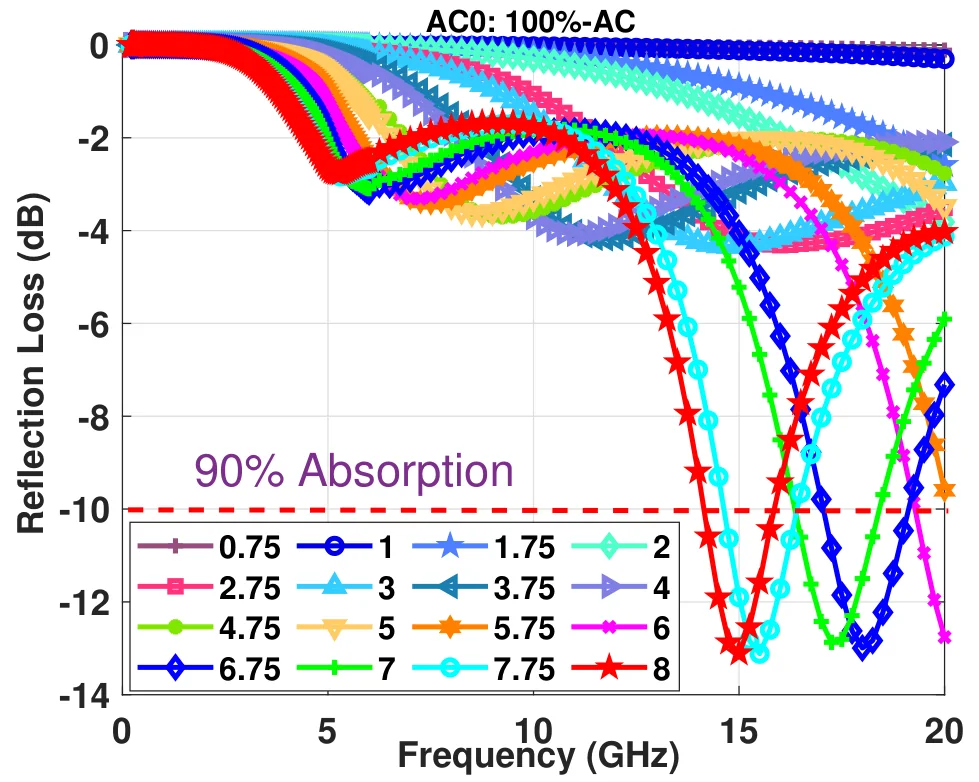
Simulated RL for AC0 composite across the 0.2–20 GHz frequency range for various sample thicknesses (ranging from 0.75 mm to 8 mm). The dashed line at −10 dB indicates 90% microwave absorption.

Figure 12 presents the RL behavior of the pure AC sample over the 0.2–20 GHz range for thicknesses from 0.75 mm to 8 mm. The results show a strong dependence of absorption performance on material thickness, following the quarter-wavelength matching principle described by (11) [12,43,49].

(11) $$d_{m}=\frac{n\lambda }{4}=\frac{nc}{4f_{m}\sqrt{\varepsilon_{r}\mu_{r}}}\;;\;n=1,\;3,\;5,\;\ldots$$

where $d_{m}$ represents the matching thickness, *n* is an odd integer (1, 3, 5, …), λ is the wavelength, *c* is the speed of light, $f_{m}$ is the matching frequency, and $\varepsilon_{r}$ and $\mu_{r}$ are the relative permittivity and permeability, respectively.

**Figure 13:**
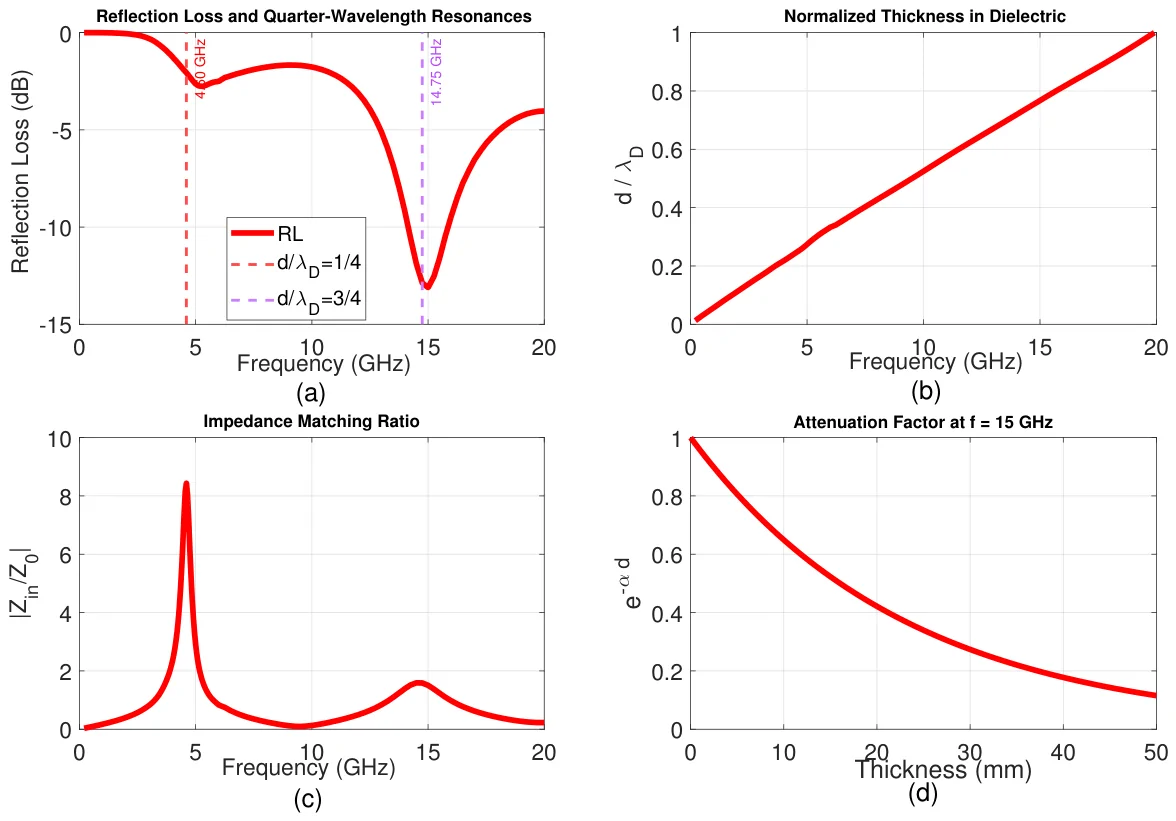
Simulated RL, impedance matching, corresponding theoretical matching thickness and attenuation constant plot of AC0 absorber at d = 8 mm.

Carbon-based microwave absorbers generally exhibit moderate permittivity and negligible magnetic permeability ($\mu_{r}\approx 1$), resulting in significant impedance mismatch with free space. Consequently, relatively large thicknesses are required to achieve effective absorption, especially at lower frequencies where long wavelengths make quarter-wavelength matching dependent on thicker materials [12]. This limitation is evident in the AC0 absorber, where thin samples show poor performance across the entire frequency range, with reflection-loss values remaining close to 0 dB. Furthermore, it is clear that the AC-based composites show weak absorption at the theoretical λ/4 positions in lower frequency ranges where simple quarter-wavelength calculations predict strong performance. In contrast, strong absorption occurs at 3λ/4 resonances at higher frequencies as illustrated in Figure 13a. The normalized electrical thickness in Figure 13b confirms that both absorption minima correspond to odd multiples of the quarter-wavelength condition. The impedance matching ratio in Figure 13c indicates substantial impedance mismatch at the fundamental resonance, whereas it approaches unity at the 3λ/4 resonance, indicating improved impedance matching. The attenuation factor in Figure 13d shows a gradual decrease in wave amplitude with propagation through the material, indicating that sufficient material thickness is required for appreciable attenuation. These results demonstrate that satisfying the quarter-wavelength condition alone is insufficient for effective absorption; favorable impedance matching and adequate attenuation within the material are also necessary. In this case, these conditions are better satisfied at the higher-order resonance, accounting for its stronger absorption.

### 5.2. Activated Carbon-Waste Filler Composites

**Figure 14:**
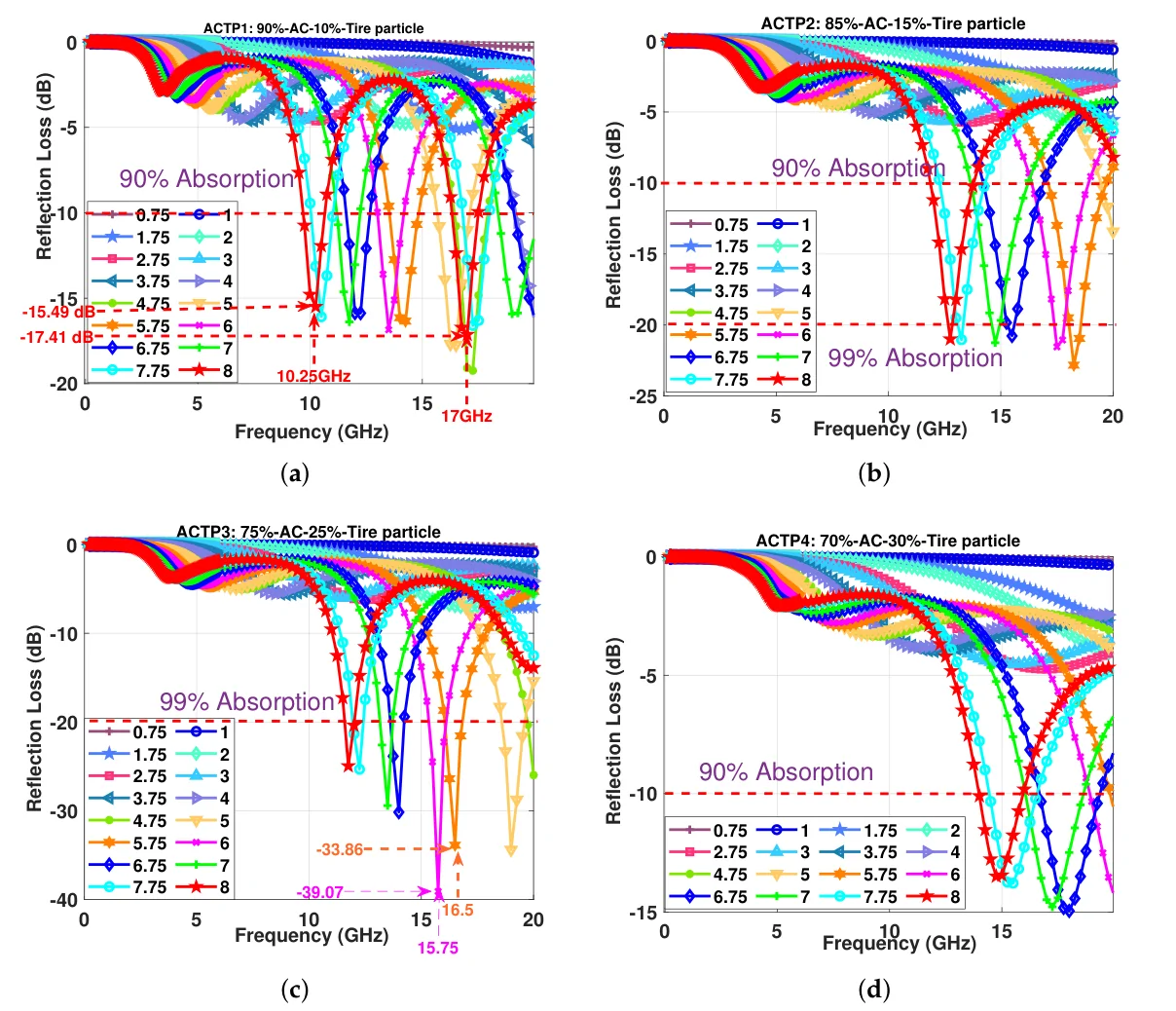
Reflection loss of the activated carbon and tire particle composites across the 0.2–20 GHz frequency range for sample thicknesses from 0.75 mm to 8 mm: (**a**) ACTP1. (**b**) ACTP2. (**c**) ACTP3. (**d**) ACTP4.

Figure 14 presents the RL behavior of the ACTP composites over the 0.2–20 GHz range for varying thicknesses. Incorporating tire particles improves absorption relative to pure activated carbon across all compositions, and the performance varies systematically with loading. At 10 and 15 wt% (ACTP1 and ACTP2), the minima deepen progressively, with several thicknesses of ACTP2 crossing the −20 dB threshold between 12 and 19 GHz, corresponding to over 99% absorption. The strongest response is obtained at 25 wt% (ACTP3), where multiple thickness configurations fall below −30 dB and the 6 mm sample reaches −39.07 dB at 15.75 GHz. Samples with thicknesses from 5 mm to 8 mm all show strong absorption, with the peaks shifting systematically to lower frequencies as thickness increases. At 30 wt% (ACTP4), the performance falls back markedly, with minima limited to approximately −13 to −15 dB, consistent with the reduced permittivity and conductivity measured for this composition. Although the ACTP composite shows improved overall performance, its resonance behavior remains similar to that of pure AC, with the dominant absorption occurring at the 3λ/4 position instead of at λ/4.

**Figure 15:**
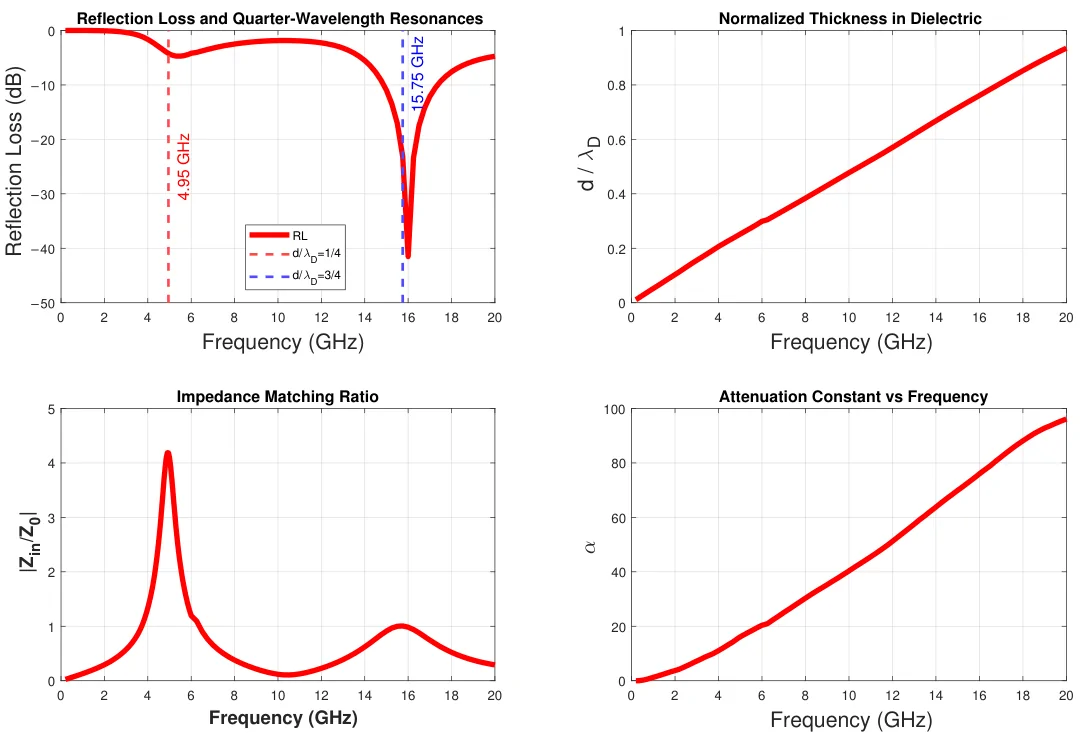
Simulated RL, impedance matching, corresponding theoretical matching thickness and attenuation constant plot of ACTP3 absorber at d = 6 mm.

Figure 15 shows the RL, normalised electrical thickness ($d/\lambda_{D}$), normalised input impedance ($|Z_{\mathrm{in}}/Z_{0}|$), and attenuation constant (α) of ACTP3 at 6 mm. Similar to AC0, ACTP3 exhibits a dominant 3λ/4 resonance. At the quarter-wavelength condition ($d/\lambda_{D}=1/4$), near 4.95 GHz, weak absorption occurs because $|Z_{\mathrm{in}}/Z_{0}|\approx 4.2$, indicating severe impedance mismatch. The relatively low attenuation constant further limits the dissipation of the electromagnetic energy entering the material, resulting in weak absorption.In contrast, near 15.75 GHz, the 3λ/4 condition coincides with $|Z_{\mathrm{in}}/Z_{0}|\approx 1$ and a higher α, enabling effective impedance matching and strong attenuation. The simultaneous achievement of favorable impedance matching and sufficient attenuation results in pronounced absorption at the higher-order resonance. This behaviour is consistent with ACTP3 exhibiting the highest $\varepsilon^{\prime \prime }$, σ, and tanδ in the ACTP series, together with a moderate $\varepsilon^{\prime }$. The high dielectric loss, associated with the connected conductive filler network observed in its SEM micrograph, provides strong electromagnetic attenuation at higher frequencies. Meanwhile, the moderate $\varepsilon^{\prime }$ helps avoid excessive surface reflection associated with high permittivity, allowing impedance matching to be achieved at the 3λ/4 resonance. These results show that satisfying the quarter-wavelength condition alone is insufficient to achieve strong absorption. Effective absorption requires an appropriate balance between $\varepsilon^{\prime }$ and dielectric loss ($\varepsilon^{\prime \prime }$ and tanδ) to achieve impedance matching and sufficient attenuation at the same frequency.

**Figure 16:**
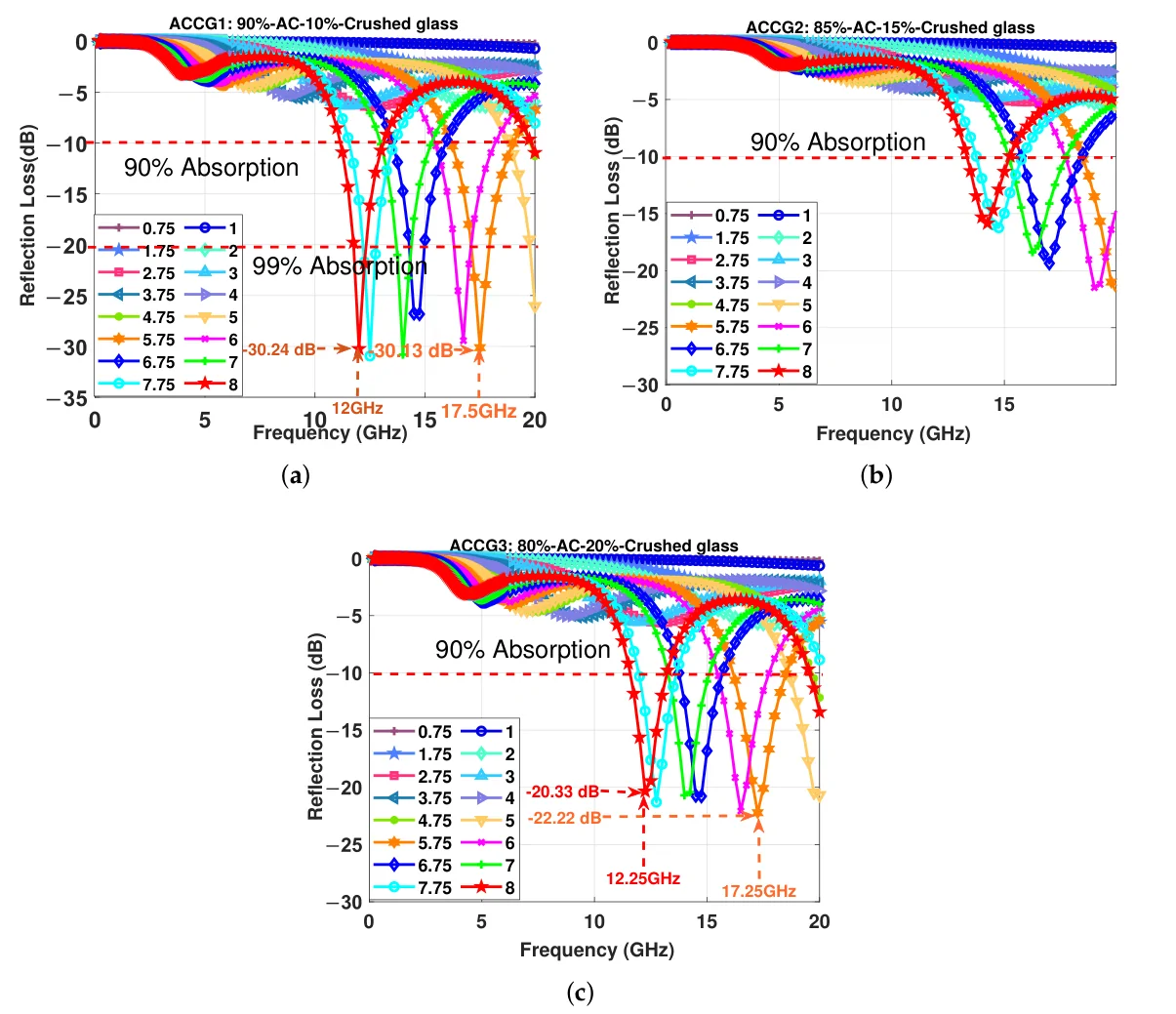
Reflection loss of the activated carbon and crushed glass composites across the 0.2–20 GHz frequency range for sample thicknesses from 0.75 mm to 8 mm: (**a**) ACCG1. (**b**) ACCG2. (**c**) ACCG3.

Figure 16 presents the reflection loss performance of the ACCG composites with crushed glass loadings of 10, 15, and 20 wt%. ACCG1 gives the strongest response of the series, reaching −30.24 dB at 12 GHz and −30.13 dB at 17.5 GHz, with several thicknesses exceeding −20 dB across the 12–20 GHz range. Increasing the glass content to 15 wt% (ACCG2) reduces peak absorption to approximately −16 to −19 dB, with most thicknesses clearing the −10 dB threshold but none reaching −20 dB. At 20 wt% (ACCG3), the performance recovers partially, with minima of −20.33 dB at 12.25 GHz and −22.22 dB at 17.25 GHz. This non-monotonic trend follows the same pattern observed in the measured permittivity, where ACCG1 shows the highest $\varepsilon^{\prime }$, ACCG2 the lowest, and ACCG3 an intermediate value. In this composition, the crushed glass acts as an insulating filler that dilutes the conductive activated carbon network and reduces the available charge-transport pathways. At the same time, the added glass introduces additional glass–carbon interfaces that promote interfacial polarization. The absorption performance across the series is therefore determined by the relative contributions of dilution and interfacial polarization, rather than by filler content alone.

The ACCG composites follow the same resonance behavior observed in the other carbon-based systems, with the dominant absorption occurring at the 3λ/4 position rather than at λ/4. The dielectric modification introduced by the crushed glass is therefore insufficient to overcome the intrinsic impedance mismatch of a non-magnetic carbon absorber, and higher-order resonances remain necessary for effective absorption.

## 6. Discussion

The present study systematically investigates activated carbon composites incorporating various functional fillers, including tire particles, and crushed glass, to examine how filler type and loading influence microwave absorption performance across the 0.2–20 GHz frequency range.

A fundamental observation across all carbon-based composites is the dominance of 3λ/4 resonance over the theoretical λ/4 resonance, as shown in Figure 13. Pure activated carbon and its composites possess moderate permittivity but negligible magnetic permeability, resulting in characteristic impedance significantly different from that of air (377 Ω). At the λ/4 condition, substantial front-surface reflection prevents adequate electromagnetic wave penetration, yielding weak absorption despite satisfying the theoretical thickness requirement. In contrast, at 3λ/4 thickness, accumulated phase shifts and multiple internal reflections create favorable impedance matching conditions ($|Z_{\mathrm{in}}/Z_{0}|\approx 1$, as in Figure 13c), enabling strong absorption. This finding aligns with the general principle that materials dominated by dielectric loss require resonance thicknesses greater than λ/4, while magnetic loss-dominated materials can achieve effective absorption at thicknesses less than λ/4 and the resonant positions shift are intrinsically related to the loss tangent of the material [12].

**Figure 17:**
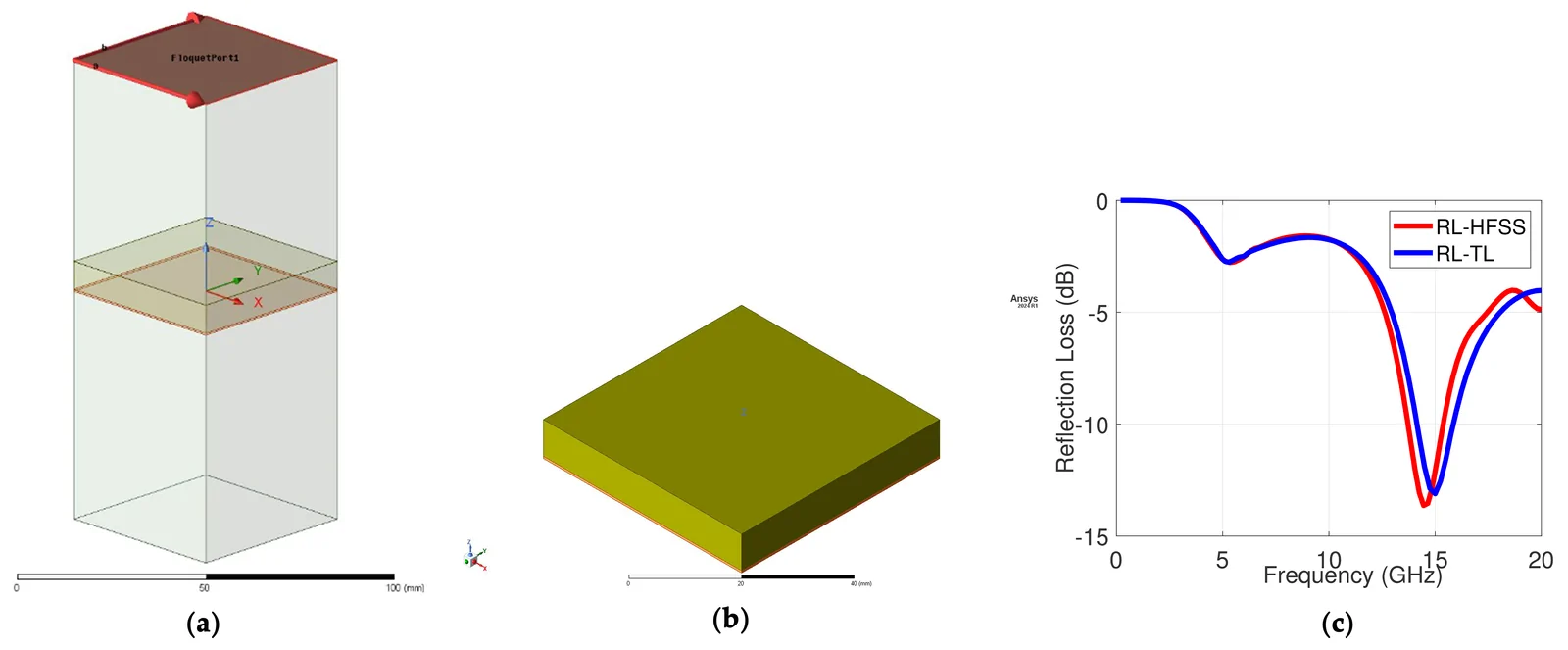
Proposedsingle-layer microwave absorbing panel: (**a**) Three-dimensional simulation model of AC0 showing the Floquet port assignment. (**b**)Top view of the AC0 absorber. (**c**) Comparison of the reflection loss of the AC0 absorber at 8 mm thickness obtained from HFSS simulation and from transmission-line calculation.

To validate the results obtained from the TL model, full-wave electromagnetic simulations were performed using Ansys HFSS. The 3D model, shown in Figure 17a, employs lattice-pair boundary conditions on the lateral faces to represent an infinite planar absorber, with a Floquet port providing plane-wave excitation at normal incidence. A perfect electric conductor (PEC) was placed behind the absorber layer to represent the metal-backed configuration and prevent transmission. The frequency-dependent complex permittivity measured with the DAK system was assigned to the absorber material through a multipole Debye dispersion model in HFSS. This model captures the variation of both the real and imaginary parts of the permittivity across multiple relaxation processes, so that the simulation reflects the material behaviour over the full frequency range. As the composites are non-magnetic, the relative permeability was set to $\mu_{r}$ = 1. Mesh convergence was assessed through adaptive mesh refinement, with a maximum change in S-parameters (ΔS) of 0.02 between successive passes used as the convergence criterion.

Single-layer AC0 absorber panel was simulated at the optimal thicknesses of 8 mm. The HFSS results agree closely with the TL calculations, as shown in Figure 17c, confirming that both analytical and full-wave approaches exhibits similar absorption behaviour. This agreement supports the 3λ/4 resonance interpretation for pure AC and indicates that the TL approach captures the essential physics despite its simplifications.

Table 2 presents a comparison of the proposed composites with recently reported waste-derived and carbon-based materials with respect to minimum RL, absorber thickness, effective bandwidth, operating frequency and filler loading. The ACTP and ACCG composites achieve RL values of −39 and −29.4 dB, respectively, at a thickness of 6 mm, with effective bandwidths of 2.5 and 2.75 GHz in the Ku-band. Several previously reported absorbers achieve substantial RL at lower thicknesses but incorporate engineered nanomaterials or magnetic constituents. For example, peanut shell/MWCNT and Fe3O4/GCs composites exhibit RL values of −28.36 and −32 dB at thicknesses of 2 and 3.5 mm, respectively. These comparisons highlight the trade-off between absorber thickness and material composition. The ground tire rubber-based composites reported in [50,51] exhibit SE values of 53.8 and 66.9 dB, respectively, demonstrating the potential of modified tire rubber for EMI shielding. However, these composites rely on CNT incorporation to enhance electrical conductivity and establish conductive pathways. In contrast, ACTP achieves an RL of −39 dB without incorporating additional conductive nanofillers or employing a porous cellular structure. This result highlights the potential of ACTP to achieve effective EMW absorption using a simpler material composition, without relying on additional conductive nanomaterials. Although SE and RL are obtained under different measurement configurations and cannot be compared quantitatively, the results demonstrate the promise of ACTP as an alternative approach to electromagnetic wave absorption using tire-derived materials.

The principal advantage of the proposed composite therefore lies in its ability to achieve substantial MWA using waste-derived constituents and conventional processing, rather than in minimizing absorber thickness. Nevertheless, the proposed composites exhibit effective absorption in the Ku-band at 6 mm and extend the absorption response across the X- and Ku-bands at 8 mm through successive higher-order resonances. Their material simplicity and use of waste-derived fillers make them promising candidates for applications such as structural panels, building cladding, and anechoic chamber linings, where absorber thickness may be less restrictive than in compact microwave devices. Further improvements could be pursued through multilayer or graded architectures designed to introduce multiple absorption resonances and broaden the effective bandwidth while reducing the total thickness. The incorporation of magnetic fillers, such as ferrites, or the integration of metamaterial structures may also improve impedance matching and absorption bandwidth, although such modifications would increase material or structural complexity.

## 7. Conclusions

This study demonstrates that low-cost, waste-derived fillers incorporated into an activated carbon matrix can achieve competitive microwave absorption while promoting sustainable materials utilization. The systematic investigation reveals fundamental insights into absorption mechanisms for carbon-based absorbers. A critical finding for activated carbon composites is the consistent dominance of 3λ/4 resonance rather than conventional λ/4 resonance due to impedance mismatch between AC materials and free space.

Among the two filler systems investigated, tire particles produced the strongest absorption performance. The ACTP3 composite, containing 25 wt% tire particles, achieved a minimum reflection loss of −39.07 dB at 15.75 GHz. In comparison, the crushed-glass composites achieved a maximum reflection loss of −30.24 dB for ACCG1 at 12 GHz. In these composites, absorption is primarily associated with Maxwell–Wagner interfacial polarisation at the interfaces between the conductive carbon and insulating glass phases. For both filler systems, filler loading was found to be a critical parameter. Excessive filler incorporation can disrupt or dilute the conductive carbon network, resulting in reduced absorption performance, as demonstrated by ACTP4 containing 30 wt% tire particles. Thus, careful optimisation of filler type, loading, and matrix composition is essential for balancing peak reflection loss, effective bandwidth, and operating frequency. Later, full-wave electromagnetic simulations performed using Ansys HFSS showed close agreement with the transmission-line calculations, further supporting the 3λ/4 resonance interpretation for the pure AC single-layer absorber. Future work should experimentally validate these predictions using fabricated panels and evaluate their mechanical and thermal durability under practical operating conditions. Further investigation of multilayer architectures may also provide opportunities to achieve enhanced broadband absorption while improving the scalability and manufacturability of the absorber system.

## Figures and Tables

**Table 1:** Composition of Activated Carbon, PVA Glue, Tire Particles, and Crushed Glass Samples.

Symbol	Activated Carbon (g)	Poly(vinyl acetate) (g)	Tire Particles (g)	Crushed Glass (g)	Filler wt% (Based on Activated Carbon)	Water (g)	Total Mass (g)
AC0	42	40.5	0	0	0	4.5	87
ACTP1	40	36	4	0	10	4	84
ACTP2	40	36	6	0	15	4	86
ACTP3	40	36	10	0	25	4	90
ACTP4	40	36	12	0	30	4	92
ACCG1	40	36	0	4	10	4	84
ACCG2	40	40.5	0	6	15	4.5	91
ACCG3	40	40.5	0	8	20	4.5	93

**Table 2:** Comparison of electromagnetic wave absorption materials.

Material	wt%	RL_min_ (dB)	Thickness	BW	Frequency Range	Ref.
			(mm)	(GHz)	(GHz)	
Apricot tree gum/FEOR	–	−71.1	1.0	3.3	12–14	[47]
Fe_3_O_4_/SiO_2_	40	−28.6	2.5	2.11	7–9.11	[52]
Peanut shell/MWCNT	60	−28.36	2	1	15–16	[53]
Coconut fibre/charcoal/epoxy	10	−25	–	–	8.2–12.4	[54]
Bamboo biochar/PE	80	48.7 (SE)	–	–	0.3–1.5	[55]
BFO750-PW	–	−17	8.0	–	2–4, 8–10, 14–16	[56]
Eggshell membrane (C@CoFe_2_O_4_)	–	−49.6	2.5	3.0	–	[57]
Fe_3_O_4_@C	50	−36	5.0	3.8	11.8–15.6	[58]
Fe_3_O_4_/GCs	30	−32	3.5	4.0	8–12	[59]
Fe_3_O_4_–Fe/G	18	−58	4.6	6.2	11.8–18	[60]
Fe_3_O_4_/C	60	−56	6.2	4.0	11–15	[61]
Fe_3_O_4_/CNT	20	−75	3.0	6.0	12–18	[62]
Fe_3_O_4_/CNT (FCC14)	30	−43	1.5	8.3	9.7–18	[63]
Carbon fibre waste/PA6 nonwoven	80 (CF)	85 (SE)	–	–	8–12	[64]
Ground tire rubber/WFPUF/CNT/CNF	20 (WCC), 4 (CNT)	53.8 (SE)	2	–	8.2–12.4	[50]
Ground tire rubber/CNT	5 (CNT)	66.9 (SE)	2.6	–	8–12	[51]
ACTP (This work)	25	−39	6	2.5	14.5–17	This work
ACCG (This work)	10	−29.4	6	2.75	16.25–19.0	This work

## Data Availability

Data will be made available upon request.
